# Oxidative stress conditions increase the frequency of *de novo* formation of the yeast [*PSI* ^+^] prion

**DOI:** 10.1111/mmi.12930

**Published:** 2015-02-11

**Authors:** Victoria A. Doronina, Gemma L. Staniforth, Shaun H. Speldewinde, Mick F. Tuite, Chris M. Grant

**Affiliations:** ^1^ Faculty of Life SciencesUniversity of ManchesterThe Michael Smith BuildingOxford RoadManchesterM13 9PTUK; ^2^ Kent Fungal GroupSchool of BiosciencesUniversity of KentCanterburyKentCT2 7NJUK

## Abstract

Prions are self‐perpetuating amyloid protein aggregates which underlie various neurodegenerative diseases in mammals and heritable traits in yeast. The molecular basis of how yeast and mammalian prions form spontaneously into infectious amyloid‐like structures is poorly understood. We have explored the hypothesis that oxidative stress is a general trigger for prion formation using the yeast [*PSI* ^+^] prion, which is the altered conformation of the Sup35 translation termination factor. We show that the frequency of [*PSI* ^+^] prion formation is elevated under conditions of oxidative stress and in mutants lacking key antioxidants. We detect increased oxidation of Sup35 methionine residues in antioxidant mutants and show that overexpression of methionine sulphoxide reductase abrogates both the oxidation of Sup35 and its conversion to the [*PSI* ^+^] prion. [*PSI* ^+^] prion formation is particularly elevated in a mutant lacking the Sod1 Cu,Zn‐superoxide dismutase. We have used fluorescence microscopy to show that the *de novo* appearance of [*PSI* *^+^*] is both rapid and increased in frequency in this mutant. Finally, electron microscopy analysis of native Sup35 reveals that similar fibrillar structures are formed in both the wild‐type and antioxidant mutants. Together, our data indicate that oxidative stress is a general trigger of [*PSI* ^+^] formation, which can be alleviated by antioxidant defenses.

## Introduction

Prions are novel, protein‐only, infectious agents associated with a group of transmissible neurodegenerative diseases typified by human Creutzfeldt Jakob disease. In mammalian cells, conversion of the normal prion protein (PrP^C^) into its infectious PrP^Sc^ conformation underlies the pathogenesis of these transmissible spongiform encephalopathies (Collinge and Clarke, 2007). This ‘protein‐only’ mechanism of infectivity also underpins the unusual inheritance of several novel epigenetic determinants found in the yeast *Saccharomyces cerevisiae* (Wickner, 1994). Around 10 different proteins are now known to form prions in yeast with many other proteins classified as potential prion candidates (Alberti *et al*., 2009). The [Het‐s] prion that controls vegetative incompatibility has also been described in *Podospora anserina*, an unrelated fungal species (Saupe, 2011) illustrating the wider occurrence of prions in fungi. Amongst the most extensively studied of the fungal prions are [*PSI* ^+^], formed from Sup35, a translation termination factor (Wickner, 1994; Tuite and Cox, 2007) and [*URE3*], the prion form of the transcriptional regulator, Ure2 (Wickner, 1994). In several cases, the infectious behaviour of the fungal prion associated with a particular phenotype has been directly demonstrated (Maddelein *et al*., 2002; King and Diaz‐Avalos, 2004; Tanaka *et al*., 2004), including [*RNQ* ^+^], the prion form of Rnq1 (Patel and Liebman, 2007), a protein of undetermined function. The [*RNQ* ^+^] prion is also one of a number of prions generically designated as [*PIN* ^+^] that are required for the *de novo* formation of [*PSI* ^+^] (Derkatch *et al*., 1996; 2001; Osherovich and Weissman, 2001). Studies on fungal prions have not only provided direct evidence for the ‘protein‐only’ nature of prion‐associated traits but also revealed how prions can contribute to the regulation of a range of cellular processes, some of which may be beneficial to the host cell (Tuite and Serio, 2010).

As with the formation of the mammalian PrP^Sc^ prion, fungal prions can also arise *de novo*; however, the underlying molecular mechanism is poorly understood in both cases. PrP^c^ is thought to adopt an alternative conformational state by spontaneous misfolding event(s) triggered possibly by mutation, mistranslation, environmental stresses and/or by disruption of the chaperone network (DeMarco and Daggett, 2005). The frequency of *de novo* formation of the [*PSI* ^+^] prion in yeast is regulated by environmental, cellular and epigenetic factors (Tuite *et al*., 2007; Tyedmers *et al*., 2008). Studies of [*PSI* ^+^] suggest that several misfolded molecules of Sup35 may spontaneously form a catalytically active oligomer that initiates prion formation (Serio *et al*., 2000). The [*PSI* ^+^] prion arises *de novo* at a frequency of ∼ 10^−5^–10^−7^ (Lund and Cox, 1981; Lancaster *et al*., 2010), but this frequency is elevated approximately 1000‐fold by overexpression of either the full length Sup35 or the prion NM domain of Sup35 (Chernoff *et al*., 1993). Several different environmental stress conditions, including oxidative stress, are capable of increasing the frequency of [*PSI* ^+^] prion formation (Tyedmers *et al*., 2008). Previously, we have demonstrated that the *de novo* formation of the [*PSI^+^*] and [*PIN* ^+^] prions is increased in yeast mutants lacking the peroxiredoxin proteins Tsa1 and Tsa2 (Sideri *et al*., 2010; 2011). Peroxiredoxins play multiple roles in protecting cells against stress; in particular, they suppress potentially harmful oxidative damage to proteins following oxidative stress. These findings implicated oxidative damage, of Sup35p, as an important trigger for the formation of the heritable [*PSI^+^*] prion in yeast.

Oxidative damage following exposure to reactive oxygen species (ROS) is a well‐established trigger of protein misfolding (Dalle‐Donne *et al*., 2003). Oxidative stress has been implicated in the *de novo* formation of mammalian prions, where elevated levels of oxidized methionine (Met) residues are detected in misfolded PrP^Sc^ relative to the native PrP^C^ form of the protein (Wolschner *et al*., 2009; Canello *et al*., 2010; Elmallah *et al*., 2013). Oxidative damage to Met residues in purified PrP^C^ has been proposed to facilitate the structural conversion underlying the sporadic formation of PrP^Sc^ (Younan *et al*., 2012). Similarly, we have shown that Met oxidation of Sup35 is elevated in *tsa1 tsa2* mutants (Sideri *et al*., 2011). Abrogating Sup35 methionine oxidation in a *tsa1 tsa2* mutant by overexpressing methionine sulphoxide reductase (MSRA) prevents [*PSI* ^+^] formation, indicating that Sup35 oxidation may underlie the switch from a soluble to an aggregated prion form of Sup35 (Sideri *et al*., 2011). Protein oxidation may therefore be a common mechanism underlying the aggregation of both mammalian and yeast *amyloidogenic proteins*.

Formation of Sup35 prion aggregates sequesters Sup35 away from its normal function of translation termination resulting in elevated levels of stop‐codon read‐through and the generation of C‐terminally extended polypeptides. It has been suggested that the shift to the [*PSI* ^+^] prion state, and the associated increase in stop‐codon read‐through, provides a mechanism for generating heritable phenotypic diversity by allowing cells to ‘reprogram’ gene expression, such that new genetic traits become uncovered that potentially aid survival through adverse conditions (Tyedmers *et al*., 2008). For example, resistance to several environmental stress conditions correlates with the [*PSI* ^+^] versus [*psi* ^−^] status of cells (Eaglestone *et al*., 1999; True and Lindquist, 2000; True *et al*., 2004). Thus, most phenotypic alterations are thought to arise due to changes in translation termination efficiency (True *et al*., 2004). In agreement with this idea, the [*PSI* ^+^] prion has now been found in a number of wild yeast strains and shown to confer diverse phenotypes that are frequently beneficial under selective conditions (Halfmann *et al*., 2012). In addition, we have previously shown that prion formation provides yeast cells with an adaptive advantage under oxidative stress conditions, since elimination of prions from *tsa1 tsa2* mutants renders the cells hypersensitive to hydrogen peroxide (Sideri *et al*., 2010).

In this study, we show that oxidation of Sup35 is a common response to oxidative stress and results in Sup35 conversion to the [*PSI* ^+^] state, presumably due to structural transitions favouring conversion to the propagatable [*PSI^+^*] form. We found the frequency of [*PSI^+^*] prion formation is elevated both in mutants lacking key antioxidants, and in response to the addition of exogenous oxidants. The increased frequency of [*PSI^+^*] formation in multiple antioxidant mutants correlates with elevated levels of methionine oxidation in Sup35, and the switch from a soluble to an aggregated form of Sup35 is suppressed by overexpressing methionine sulphoxide reductase. We have used fluorescence microscopy in conjunction with Sup35NM‐GFP and electron microscopy (EM) analysis of native Sup35 to characterize Sup35 aggregate formation and show that similar fibrillar structures are formed in wild‐type and antioxidant mutants. To our knowledge, this is the first systematic investigation of the role of oxidative stress in yeast prion formation.

## Results

### [*PSI* ^+^] prion formation is increased in response to oxidative stress

**Figure 1:**
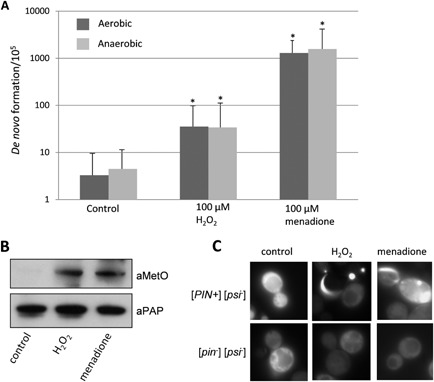
Oxidative stress conditions increase the frequency of [*PSI* ^+^] prion formation. A. [*PSI* ^+^] prion formation was quantified using an engineered *ura3–14* allele, which contains the *ade1–14* nonsense mutation inserted into the wild‐type *URA* *3* gene (Manogaran *et al*., 2006). [*PSI* ^+^] prion formation was scored by growth on media lacking uracil, indicative of decreased translational termination efficiency. [*PSI* ^+^] formation was also scored by growth on media lacking uracil under anaerobic conditions. [*PSI* ^+^] formation was differentiated from nuclear gene mutations which give rise to uracil prototrophy by their irreversible elimination in GdnHCl. The control [*PIN* ^+^] [*psi* ^−^] strain was grown in the presence of 100 μM hydrogen peroxide or 100 μM menadione for 16 h prior to scoring [*PSI* ^+^] prion formation (in the absence of exogenous oxidants). Data shown are the means of at least three independent experiments expressed as the frequency of [*PSI* ^+^] prion colonies per 10^5^ viable cells. Error bars denote the standard deviation. *P < 0.05. B. Western blot analysis of Sup35 oxidation. Sup35 was affinity purified using TAP chromatography from a control [*PIN* ^+^] [*psi* ^−^] strain following growth in the presence of 100 μM hydrogen peroxide or 100 μM menadione for 16 h. Western blots were probed with α‐PAP to confirm that similar amounts of Sup35 were purified from each strain. Sup35 oxidation was detected using antibodies that recognize methionine sulphoxide (αMetO). C. Representative fluorescence micrographs are shown for the [*PIN* ^+^] [*psi* ^+^] control strain containing the Sup35NM‐GFP plasmid following growth in the presence of 100 μM hydrogen peroxide or 100 μM menadione for 16 h. The Sup35NM‐GFP plasmid was induced for 2 h using copper prior to visualizing aggregate formation.

To assay for [*PSI* ^+^] formation, we used a *ura3–14* allele containing the *ade1–14* nonsense mutation engineered into the wild‐type *URA3* gene (Manogaran *et al*., 2006). The *ura3–14* allele allows [*PSI* ^+^] prion formation to be scored by growth on medium lacking uracil, indicative of decreased translational termination efficiency in [*PSI* ^+^] cells. [*PSI* ^+^] formation was differentiated from nuclear gene mutations which give rise to uracil prototrophy by their irreversible elimination in 3 mM guanidine hydrochloride (GdnHCl) (Tuite *et al*., 1981). This simple diagnostic assay has been widely used to identify prion‐associated traits including [*PSI* ^+^] and [*PIN* ^+^] (e.g. Derkatch *et al*., 2001; Ganusova *et al*., 2006; Tyedmers *et al*., 2008; Halfmann *et al*., 2012). Using this assay, the frequency of *de novo* [*PSI* ^+^] prion formation in a [*PIN* ^+^][*psi* ^−^] strain was estimated to be approximately 5 × 10^−5^ (Fig. 1A) comparable with previously reported frequencies ranging between ∼ 10^−5^ and 10^−7^ (Lund and Cox, 1981; Lancaster *et al*., 2010).

We tested whether oxidative stress caused by the addition of exogenous oxidants increases the frequency of [*PSI* ^+^] prion formation. The control [*PIN* ^+^][*psi* ^−^] strain was grown in the presence of 100 μM hydrogen peroxide or the superoxide anion generator menadione for 16 h prior to scoring [*PSI* ^+^] prion formation. These concentrations of oxidants are quite mild and only moderately slow the growth of a wild‐type strain. Growth in the presence of 100 μM hydrogen peroxide increased the frequency of [*PSI* ^+^] prion formation by approximately 10‐fold (Fig. 1A). Stronger induction of [*PSI* ^+^] prion formation was observed in response to the superoxide anion, generated by exposure to menadione, which increased the frequency of formation by approximately 300‐fold in response to 100 μM menadione exposure. To confirm that *de novo* [*PSI* ^+^] prion formation occurs during the 16 h exposure to the oxidant rather than during the subsequent growth on selective plates used to score for [*PSI* ^+^] prion formation, plates were also incubated under anaerobic conditions. No differences in the frequency of [*PSI* ^+^] prion formation following exposure to hydrogen peroxide or the superoxide anion were observed when cells were grown on plates under aerobic compared with anaerobic conditions (Fig. 1A). These data indicate that the oxidant‐induced *de novo* [*PSI* ^+^] prion formation scored in these experiments occurs during exposure to the oxidants in the initial 24 h growth period.

### Increased frequency of *de novo* [*PSI* ^+^] prion formation is a common feature of antioxidant mutants

**Figure 2:**
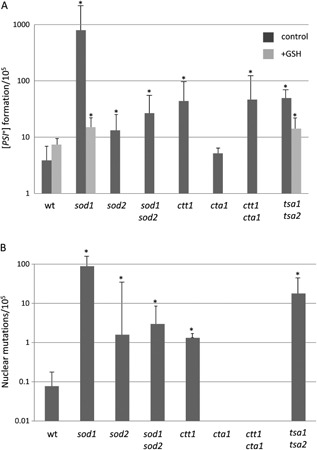
The frequency of [*PSI* ^+^] formation is increased in antioxidant mutants. A. [*PSI* ^+^] prion formation was quantified in [*PIN* ^+^] [*psi* ^−^] versions of the indicated antioxidant mutant strains as described for Fig. 1. The addition of 1 mM GSH during the initial 16 h growth period reduced the high frequency of [*PSI* ^+^] prion formation observed in the *sod1* and *tsa1 tsa2* mutants to a level approaching that of the wild‐type strain. B. The nuclear mutation rate was quantified by the formation of Ura ^+^ colonies which are not curable with GdnHC. Data shown are the means of at least three independent experiments expressed as the number of colonies per 10^5^ viable cells. Error bars denote the standard deviation. *P < 0.05.

The *ura3–14* reporter plasmid was introduced into yeast mutants lacking major antioxidants, including superoxide dismutases (*sod1*, *sod2*), catalases (*ctt1*, *cta1*) and peroxiredoxins (*tsa1*, *tsa2*). The frequency of *de novo* [*PSI* ^+^] prion formation was significantly increased in *tsa1 tsa2* mutants (Fig. 2A), as previously described (Sideri *et al*., 2010; 2011). Similarly, the frequency of [*PSI* ^+^] prion formation was elevated by approximately 10‐fold in mutants lacking *CTT1*, encoding cytosolic catalase. In contrast, loss of peroxisomal *CTA1* did not significantly affect [*PSI* ^+^] prion formation and no further elevation was observed in a double *ctt1 cta1* mutant. Loss of *SOD1*, encoding cytosolic copper–zinc superoxide dismutase, caused the most dramatic effect, increasing the frequency of *de novo* [*PSI* ^+^] prion formation almost 200‐fold. Mutants lacking the Sod2 mitochondrial manganese superoxide dismutase showed an increased frequency of [*PSI* ^+^] prion formation of approximately fourfold, but only a modest further increase was observed in a double *sod1 sod2* mutant.

Given the increased frequency of [*PSI* ^+^] prion formation observed in antioxidant mutants, we tested whether the addition of exogenous glutathione (GSH) could prevent prion formation in antioxidant mutants. GSH is an essential metabolite that protects cells against oxidative stress (Schafer and Buettner, 2001). Exogenous GSH can enter yeast cells and provide protection against oxidative stress (Grant *et al*., 1996). GSH addition reduced the high frequency of [*PSI* ^+^] prion formation observed in the *sod1* and *tsa1 tsa2* mutants to a level approaching that of the wild type, indicating that the increased frequency of prion formation caused by oxidative stress could be abrogated by addition of an exogenous antioxidant.

The nuclear mutation rate was scored in these antioxidant mutants by the formation of Ura^+^ colonies that are not curable with GdnHCl (Fig. 2B). This is to control for any mutations that stabilize nonsense mRNAs, or any mutations in termination factors (Sup35, Sup45) or the translation apparatus itself that might also cause read‐through at the *ura3–14* stop codon. The mutants that showed the highest frequencies of [*PSI* ^+^] prion formation also tended to have higher frequencies of nuclear mutations. This ranged from approximately 1 × 10^−3^ in the *sod1* mutant to 1 × 10^−5^ in the *ctt1* mutant. However, nuclear mutations were formed at significantly lower frequencies compared with the frequency of *de novo* [*PSI* ^+^] prion formation. Taken together, these data indicate that increased [*PSI* ^+^] prion formation is a common feature of antioxidant mutants.

### Sup35 methionine oxidation is increased in antioxidant mutants

**Figure 3:**
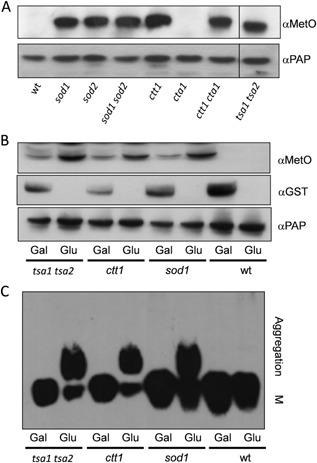
Overexpression of *MSRA* protects Sup35 against methionine oxidation and prion formation. A. Sup35 was affinity purified using TAP chromatography from a wild‐type strain and the indicated antioxidant mutants. Western blots were probed with α‐PAP to confirm that similar amounts of Sup35 were purified from each strain. Sup35 oxidation was detected using antibodies that recognize methionine sulphoxide (αMetO). B. Methionine sulphoxide reductase (*MSRA*) was overexpressed using plasmid *GAL1* *‐* *MSRA* *‐* *GST* in wild‐type and antioxidant mutant strains. Overexpression was confirmed under inducing (Gal) versus repressing (Glu) conditions using an anti‐GST antibody (αGST). *MSRA* expression prevented methionine oxidation of Sup35 detected using the α‐MetO antibody. C. SDS‐resistant Sup35 aggregates were detected in the same strains using SDD‐AGE. Aggregate and monomer (M) forms are indicated.

We have previously shown that loss of *TSA1* and *TSA2* results in elevated levels of Sup35 methionine oxidation (Sideri *et al*., 2011). Abrogating Sup35 methionine oxidation by overexpressing MSRA prevented [*PSI* ^+^] formation, indicating that Sup35 oxidation may underlie the switch from a soluble to an aggregated prion form of Sup35 in *tsa1 tsa2* mutants. We therefore examined whether Sup35 is similarly oxidized in other antioxidant mutants by immunoblot analysis using an antibody that recognizes methionine sulfoxide (MetO). The basal levels of MetO were significantly elevated in antioxidant mutants, which showed an increased rate of [*PSI* ^+^] prion formation, including *sod1*, *sod2*, *sod1 sod2*, *ctt1*, *ctt1 cta1* and *tsa1 tsa2* mutants (Fig. 3A). MetO formation was not detected in Sup35 in the wild‐type strain, nor in a *cta1* mutant strain which did not significantly increase the frequency of [*PSI* ^+^] prion formation.

If methionine oxidation underlies the switch to the [*PSI* ^+^] prion, we reasoned that MetO should also be detected in Sup35 from cells exposed to oxidative stress condition that promote prion formation. We therefore examined Sup35 from a control [*PIN* ^+^][*psi* ^−^] strain grown in the presence of 100 μM hydrogen peroxide or the superoxide anion generator menadione for 16 h as described in Fig. 1A. These oxidative stress conditions increased the frequency of [*PSI* ^+^] prion formation (Fig. 1A) and also significantly increased MetO levels.

**Figure 4:**
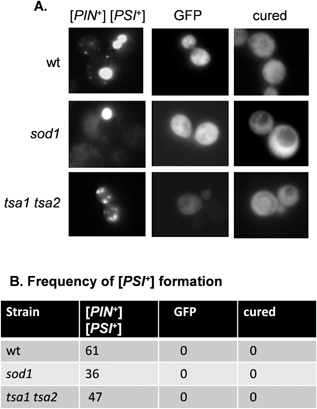
Visualization of [*PSI* ^+^] prion formation in antioxidant mutants. A. Representative fluorescence micrographs are shown for [*PIN* ^+^] [*PSI* ^+^] versions of the wild‐type, *sod1* and *tsa1 tsa2* mutant strains containing the Sup35NM‐GFP plasmid. The Sup35NM‐GFP plasmid was induced for 2 h using copper prior to visualizing aggregate formation. Cured strains were analyzed following growth with 3 mM GndHCl. A control GFP plasmid (GFP) resulted in diffuse cytoplasmic fluorescence in all strains examined ruling out any non‐specific effects on protein aggregation in these mutants. B. The percentage of cells containing visible puncta is shown for each strain from an average of 200 cells counted.

To determine whether methionine oxidation correlates with Sup35 aggregation, we examined whether overexpression of MSRA could protect against methionine oxidation and prevent [*PSI* ^+^] prion formation. Wild‐type, *ctt1*, *sod1* and *tsa1 tsa2* mutant strains were first transformed with a *GAL1‐MSRA‐GST* plasmid and then [*pin* ^−^][*psi* ^−^] derivatives generated by growth with 3 mM GdnHCl. The resulting [*pin* ^−^][*psi* ^−^] transformed colonies were grown in SD or SGal media for approximately 40 generations in liquid culture to allow for the formation of new prions. As expected, no MSRA expression was observed in glucose grown cells and elevated MetO formation was detected in the antioxidant mutants (Fig. 3B). Growth on galactose was confirmed to induce the expression of *MSRA*. Elevated MSRA *correlated with* decreased levels of methionine oxidation in the *ctt1*, *sod1* and *tsa1 tsa2* mutants (Fig. 4B). Semi‐denaturing detergent–agarose gel electrophoresis (SDD–AGE) was used to examine whether methionine oxidation influences the formation of high molecular weight sodium dodecyl sulfate (SDS)‐resistant Sup35 aggregates, diagnostic of [*PSI* ^+^] prion formation. High‐molecular weight SDS‐resistant aggregates of Sup35 were observed in *ctt1*, *sod1* and *tsa1 tsa2* mutants where *MSRA* expression was repressed by growth on glucose (Fig. 3C). In contrast, these aggregates did not form when *MSRA* was overexpressed by growth on galactose (Fig. 3C). These data indicate that methionine oxidation in Sup35 plays a critical role in the *de novo* formation of the [*PSI* ^+^] prion in antioxidant mutants.

### Visualization of Sup35 aggregation in antioxidant mutants

We used a *SUP35NM‐GFP* fusion protein to visualize Sup35 aggregate formation as previously described (Patino *et al*., 1995). This construct contains the amino‐terminal prion forming domain of Sup35 under the control of the copper regulatable *CUP1* promoter (*SUP35NM‐GFP*). Our studies focused on the *sod1* mutant since it has the highest rate of prion formation detected in any of the antioxidant mutants examined. Following induction with copper, diffuse cytoplasmic fluorescence is observed in [*psi* ^−^] cells, whereas coalescence of newly made Sup35‐GFP with pre‐existing Sup35 aggregates in [*PSI* ^+^] allows the detection of [*PSI* ^+^] foci. The Sup35‐GFP reporter construct was introduced into [*PIN* ^+^][*PSI* ^+^] versions of the *sod1* and *tsa1 tsa2* mutant strains, which were grown for 16 h prior to inducing the expression of *SUP35‐GFP* for 2 h (Fig. 4A). Many large aggregates of Sup35 were detected in the majority of [*PIN* ^+^][*PSI* ^+^] control cells following the 2 h induction period (Fig. 4). Similarly, Sup35‐GFP was detected predominantly as large bright fluorescent foci in approximately 36% of *sod1* mutant cells, comparable with the [*PIN* ^+^][*PSI* ^+^] control strain. In contrast, the foci were of varying sizes in *tsa1 tsa2* mutant cells. We ruled out any non‐specific effects on protein aggregation in these mutants since expression of a control GFP protein resulted in diffuse cytoplasmic fluorescence in all strains examined (Fig. 4). One genetic criterion for a yeast prion is reversible curability (Wickner, 1994). GdnHCl blocks the propagation of yeast prions by inhibiting Hsp104, a molecular chaperone that is absolutely required for yeast prion propagation (Ferreira *et al*., 2001; Jung and Masison, 2001). Treatment of all the [*PIN* ^+^][*PSI* ^+^] strains with GdnHCl resulted in diffuse cytoplasmic Sup35‐GFP fluorescence, with no detectable foci, confirming the requirement for Hsp104 to propagate [*PSI^+^*] prion formation in these strains (Fig. 4).

**Figure 5:**
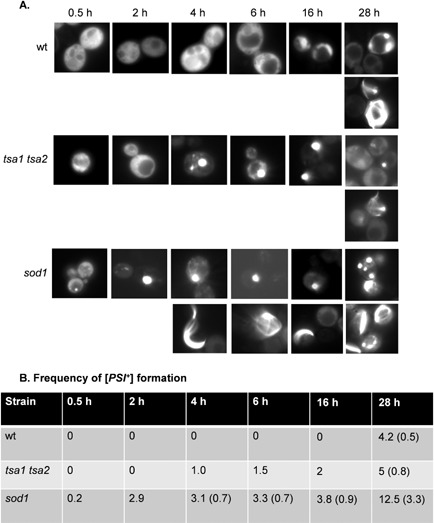
A [*PIN* ^+^] [psi ^−^] sod1 mutant shows increased frequency and rate of [*PSI* ^+^] prion formation. A. Fluorescence micrographs are shown for [*PIN* ^+^] [*psi* ^−^] versions of the wild‐type, *sod1* and *tsa1 tsa2* mutant strains containing the Sup35NM‐GFP plasmid induced with copper for the indicated times. Representative images are shown where puncta formation was first detected in the wild‐type (28 h), *sod1* (0.5 h) and *tsa1 tsa2* (4 h) strains (upper rows of images). Representative images are shown where rod‐ and ribbon‐like aggregates, indicative of *de novo* prion formation, were detected in the wild‐type (28 h), *sod1* (4 h) and *tsa1 tsa2* (28 h) strains (lower rows of images). B. The percentage of cells containing visible puncta and rod‐ and ribbon‐like aggregates (in parentheses) is shown for each strain from an average of 500 cells counted. The numbers indicate the percentage of cells containing puncta at each time point. Numbers in parentheses are the percentage of cells containing rod‐ or ribbon‐like aggregates.

Overexpression of Sup35 in [*PIN* ^+^][*psi* ^−^] cells leads to the *de novo* appearance of the [*PSI^+^*] prion since the excess Sup35 increases the possibility for prion seed formation (Wickner, 1994). We found that overexpression of Sup35‐GFP in a [*PIN* ^+^][*psi* ^−^] control strain resulted in detectable protein aggregates following 28 h of expression induced by copper addition (Fig. 5), similar to previous reports (Bradley and Liebman, 2004; Mathur *et al*., 2010). The detectable aggregates included large fluorescent aggregates that arise due to the ‘decoration’ of existing aggregates, as well as rod‐ and ribbon‐like aggregate characteristics of the *de novo* formation of [*PSI^+^*]. We examined the formation of aggregates in antioxidant mutants to determine whether fluorescent foci are detectable at earlier time points. Multiple large foci were detected in approximately 1% of *tsa1 tsa2* mutant cells examined within 4 h, but again rod‐ and ribbon‐like aggregates could only be detected within 28 h (Fig. 5). In comparison, small foci were detected in the *sod1* mutant following as little as 30 min expression of the Sup35‐GFP construct (Fig. 5). By 4 h, more than 3% of *sod1* mutant cells examined contained large fluorescent foci. Rod‐ and ribbon‐like aggregate characteristics of the *de novo* formation of [*PSI^+^*] could also be detected within as little as 4 h in the *sod1* mutant. These aggregates were confirmed to be [*PSI^+^*] prions since they were curable with GdnHCl. Loss of antioxidants, therefore, both increases the frequency of [*PSI^+^*] appearance as well as shortens the time in which detectable aggregates are formed.

To further confirm oxidative stress conditions promote [*PSI^+^*] formation, we used the Sup35‐GFP reporter to visualize Sup35 aggregate formation following exposure to oxidants. The control [*PIN* ^+^][*psi* ^−^] strain was grown in the presence of 100 μM hydrogen peroxide or the superoxide anion generator menadione for 16 h as described in Fig. 1A. Following induction of *SUP35‐GFP* for 2 h, both puncta and rod‐ and ribbon‐like aggregates could be detected in the cells exposed to hydrogen peroxide or menadione, indicative of [*PSI^+^*] formation (Fig. 1C).

### Formation of Sup35 fibrils in antioxidant mutants

**Figure 6:**
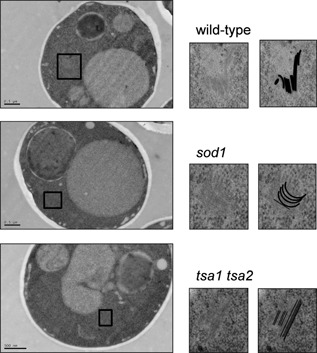
EM images of [*PSI* ^+^] aggregates in antioxidant mutants. Representative EM micrographs are shown for [*PIN* ^+^] [*PSI* ^+^] versions of the wild‐type, sod1 and tsa1 tsa2 mutant strains. The boxed areas in the main images are magnified on the right along with images containing line drawings of each aggregate.

Prion formation in *sod1* mutants has some unusual features relative to wild type including a high frequency of *de novo* prion formation and a fast appearance of rod‐ and ring‐like aggregate structures. We therefore used EM to further visualize the aggregate structures formed by Sup35 in antioxidant mutants to determine whether they show any differences to the structures formed in control [*PSI* ^+^] strains. Overexpression of *NM‐GFP* has previously been used to facilitate visualization of the fibrillar structures of [*PSI* ^+^] formed inside live yeast cells (Kawai‐Noma *et al*., 2010; Tyedmers *et al*., 2010; Saibil *et al*., 2012). We were able to detect similar structures formed in the [*PSI* ^+^] control strain and [*PSI* ^+^] versions of *tsa1 tsa2* and *sod1* mutants (Fig. 6). General protein aggregation would be expected to be detected as amorphous, irregular structures using EM. Instead, we observed the formation of spherical structures with ordered parallel fibrils in the control and antioxidant mutant strains, suggesting the presence of a similar amyloid fiber organization in these mutants. In all cases, curing with GdnHCl generated [*pin* ^−^][*psi* ^−^] cells which contained no visible fibrillar structures (data not shown).

## Discussion

Peroxiredoxins (Prx) have multiple roles in stress protection, acting as antioxidants, molecular chaperones and in the regulation of signal transduction (Wood *et al*., 2003). Tsa1 is the major yeast 2‐Cys Prx and acts as an antioxidant in the detoxification of hydroperoxides (Garrido and Grant, 2002; Wong *et al*., 2004), particularly as a result of endogenous ROS generated during normal aerobic metabolism (Iraqui *et al*., 2009). The Tsa2 peroxiredoxin is highly homologous to Tsa1 (86% identity) and possesses similar peroxidase activity, although it is normally expressed at significantly lower levels compared with Tsa1 (Jang *et al*., 2004; Wong *et al*., 2004). We previously showed that a *tsa1 tsa2* mutant of 74D‐694 gives rise to [*PSI^+^*] prion strains at a high frequency (Sideri *et al*., 2010). In this current study, we have quantified prion formation using a *ura3–14* allele‐based assay and show that [*PSI^+^*] prion formation is elevated by approximately 11‐fold in a *tsa1 tsa2* mutant compared with a wild‐type strain. Given the multiple roles that Prx's play in stress defense, it was unclear if elevated prion formation is a common response to oxidative stress conditions. Our current data indicate that prion formation is similarly increased in a range of antioxidant mutants, as well as in response to the addition of exogenous oxidants suggesting that protein oxidative damage may be a common mechanism underlying the switch from a normal soluble protein to the amyloid prion form of Sup35.

We found that oxidative stress induced by exposure to hydrogen peroxide or menadione increased the frequency of [*PSI^+^*] prion formation. Exposure to 100 μM hydrogen peroxide increased the frequency of [*PSI^+^*] prion formation by approximately 10‐fold compared with a 300‐fold increase in response to menadione. Menadione is a redox‐cycling agent which transfers electrons to molecular oxygen to generate the superoxide anion, and hence it is difficult to directly compare its dose–response effects on prion formation with that of hydrogen peroxide. H_2_O_2_ is a ubiquitous molecule formed as a byproduct of aerobic respiration and following exposure to diverse biological and environmental factors. It must be removed from cells to avoid Fenton and Haber–Weiss reactions, leading to the formation of highly reactive hydroxyl radicals (OH^·^). The superoxide anion (O_2_^−^) is generated by one electron reduction of oxygen and can be produced from the mitochondrial electron transport chain. The superoxide anion can be dismutated to hydrogen peroxide by superoxide dismutases and can generate hydroxyl radicals via metal‐catalyzed reactions. Hence, hydrogen peroxide and the superoxide anion would be expected to oxidize amino acid residues through the generation of the hydroxyl radical (Berlett and Stadtman, 1997).

Superoxide dismutases (SODs) are ubiquitous antioxidants which convert the superoxide anion to hydrogen peroxide. Yeast contains a cytoplasmic Cu,Zn‐SOD (Sod1) and a mitochondrial matrix Mn‐SOD (Sod2) which play distinct roles during oxidative stress conditions (Culotta *et al*., 2006). Cells deleted for *SOD1* are hypersensitive to the superoxide anion and display a number of oxidative stress‐related phenotypes including vacuole damage and increased free iron concentrations (Culotta, 2000). Mutants deleted for *SOD2* are less affected in growth and stress sensitivity compared with *sod1* mutants but do show a reduced ability to grow under respiratory conditions (van Loon *et al*., 1986). Interestingly, loss of *SOD1* caused a dramatic increase in [*PSI^+^*] prion formation, which was elevated by approximately 200‐fold. In contrast, a modest fourfold increase in [*PSI^+^*] prion formation was detected in a *sod2* mutant. This difference in prion formation may reflect the difference in superoxide sensitivity between the *sod1* and *sod2* mutants, with the Sod1 playing the predominant role in protection against endogenous superoxide. It is unclear why *sod1 sod2* mutants display reduced [*PSI^+^*] prion formation rates compared with a *sod1* single mutant, but this may indicate that compensatory changes occur in the double mutant which reduce prion formation.

Catalases are heme‐containing enzymes that catalyze the dismutation of H_2_O_2_ into H_2_O and O_2_. Yeast expresses a peroxisomal catalase A encoded by *CTA1* and a cytosolic catalase T encoded by *CTT1*. Ctt1 is thought to play a more general role as an antioxidant during exposure to oxidative stress, whereas Cta1 functions in the detoxification of H_2_O_2_ generated from fatty acid β‐oxidation (Martinez‐Pastor *et al*., 1996). [*PSI^+^*] prion formation was elevated approximately 10‐fold in *ctt1* and *ctt1 cta1* mutants, but loss of *CTA1* did not appear to affect prion formation. This is in agreement with Ctt1 protecting against endogenous hydrogen peroxide and therefore required to suppress [*PSI* ^+^] during normal growth conditions, compared with Cta1 which plays a more specialized role in peroxisomes. Thus loss of both superoxide dismutases and catalases, which are major antioxidants in eukaryotic cells, both increased the frequency of *de novo* formation of the [*PSI^+^*] prion, suggesting that prion formation is a common response in cells subjected to oxidative stress.

Reactive oxygen species are toxic molecules that can potentially oxidize all amino acids in proteins, including Met residues, which are particularly susceptible to oxidation by ROS (Dean *et al*., 1997). We previously detected elevated levels of methionine oxidation in *tsa1 tsa2* mutants (Sideri *et al*., 2011), and similarly, MetO levels were found to be increased in *sod1*, *sod2*, *sod1 sod2*, *ctt1* and *ctt1 cta1* mutants, which all display increased [*PSI^+^*] prion formation. A similar increase in the levels of Mets–SO formation was observed in all of these mutants, despite the finding that they showed significant differences in the frequency of *de novo* [*PSI^+^*] prion formation. This may indicate that methionine oxidation is not the most important form of protein oxidative damage for prion formation. Alternatively, the Western blots used to detect MetO levels may not provide a quantitative measure of methionine oxidation. Nevertheless, no increase in MetO levels was detected in *cta1* mutants, which was unaffected in prion formation. We previously showed that overexpression of MSRA prevents MetO formation in a *tsa1 tsa2* mutant and largely prevented the conformational shift from soluble Sup35 to the insoluble aggregated form (Sideri *et al*., 2011). Similarly, overexpression of MSRA in antioxidant mutants protected against methionine oxidation in these mutants and abrogated Sup35 aggregation. These data confirm that methionine oxidation of Sup35 plays a critical role in the *de novo* formation of [*PSI^+^*] during oxidative stress conditions.

Since the spontaneous frequency of [*PSI^+^*] prion formation is so low, *de novo* formation is often visualized by overexpression of Sup35, as overexpression of Sup35 causes very high rates of [*PSI^+^*] formation (Derkatch *et al*., 1997). Overexpression of *SUP35NM‐GFP* in [*PIN* ^+^][*psi* ^−^] cells facilitates the detection of ring‐, rod‐ and ribbon‐like aggregates. These ring‐like structures are found in the cell periphery or surrounding the vacuole and mature into an infectious prion state detected as large dot‐like aggregates (Zhou *et al*., 2001; Ganusova *et al*., 2006). Overexpression of *SUP35NM‐GFP* in [*PIN* ^+^][*PSI* ^+^] cells can give rise to cells with multiple large fluorescent puncta, where Sup35‐GFP decorates pre‐existing aggregates. We found that overexpression of *SUP35NM‐GFP* for 2 h in [*PIN* ^+^][*PSI* ^+^] versions of *sod1* and *tsa1 tsa2* mutant cells gave rise to multiple large fluorescent foci. The foci detected in the *tsa1 tsa2* mutant were more variable in size relative to the control [*PIN* ^+^][*PSI*+] strain and *sod1* mutant. Overexpression of *SUP35NM‐GFP* in [*PIN* ^+^][*psi* ^−^] versions of *sod1* and *tsa1 tsa2* mutant cells gave rise to detectable fluorescent foci at much earlier time points than in the control [*PIN* ^+^][*psi* ^−^] strain. This was most apparent in the *sod1* mutant, where small foci were detected within 30 min and larger foci were detected in more than 3% of cells examined following 4 h of overexpression of *SUP35NM‐GFP*. Large foci were also detected in approximately 1% of *tsa1 tsa2* mutant cells following overexpression of *SUP35NM‐GFP* for 4 h. In contrast, large fluorescent puncta were only detected in the control [*PIN* ^+^][*psi* ^−^] strain within 28 h of overexpression of *SUP35NM‐GFP*. Rare, ring‐ and rod‐like structures, which are thought to represent *de novo* [*PSI* ^+^] formation, were also detected at this time point. In contrast, ring‐ and rod‐like structures could be detected within 4 h in the *sod1* mutant. Thus, the high frequency of [*PSI* ^+^] prion formation in *sod1* mutants correlates with the earlier *de novo* appearance of [*PSI* ^+^] prion aggregates.

Overexpression of *SUP35NM‐GFP* in [*PIN* ^+^][*PSI* ^+^] cells gives rise to cells with multiple large aggregates that have been characterized using EM (Kawai‐Noma *et al*., 2010; Tyedmers *et al*., 2010; Saibil *et al*., 2012). Analysis of native Sup35 in *sod1* and *tsa1 tsa2* mutants revealed the presence of similar spherical structures with ordered parallel fibrils, which are comparable with a control strain. Additionally, similar SDS‐resistant high molecular weight aggregates were detected using SDD–AGE analysis in wild‐type and antioxidant mutants. Thus, although the frequency of [*PSI* ^+^] formation is elevated during oxidative stress conditions, there does not appear to be any obvious structural differences in the protein aggregates formed under these conditions. Oxidative stress is a major biological problem that has proposed causal relationships with many disease processes including cancer, neurodegenerative disease and cardiovascular disease (Gutteridge, 1993). All organisms are exposed to ROS during the course of normal aerobic metabolism or following exposure to radical‐generating compounds. In addition, there are many established links between oxidative stress and aging, and, for example, ROS may play a causal role in cellular decline during aging (Harman, 1972). The molecular basis by which mammalian and fungal prions arise spontaneously is poorly understood at present. Our data are therefore important since they indicate a potential causal link for oxidative protein damage, in the switch from a normal soluble protein to the amyloid form.

Loss of yeast *SOD1* had the most dramatic effect on [*PSI* ^+^] formation, increasing the frequency of prion formation by approximately 200‐fold when compared with the wild‐type strain. Additionally, overexpression of *SUP35NM‐GFP* in a [*PIN* ^+^][*psi* ^−^] strain resulted in the detection of prion‐like structures immediately after expression. The observed puncta were most likely aggregates that had already formed in cells, and were decorated with newly expressed Sup35 following induction of *SUP35NM‐GFP*. Moreover, rod‐ and ring‐like structures, which are thought to represent the initial stages in *de novo* prion formation (Mathur *et al*., 2010), were detected in *sod1* mutants after 4 h of *SUP35NM‐GFP* expression, compared with 28 h in other strains. In other work, we have shown that the frequency of *de novo* formation of the [*PIN* ^+^] prion is similarly increased in a *sod1* mutant strain (data not shown). Thus, two sequence‐unrelated yeast prions, [*PSI* ^+^]/Sup35 and [*PIN* ^+^]/Rnq1, are similarly formed in a *sod1* mutant, suggesting that prion formation may be a common phenomenon in this mutant. Mutations in human *SOD1* have been associated with amyotrophic lateral sclerosis (ALS), which is a fatal neurodegenerative disease (Sreedharan and Brown, 2013). Hence, our data may also indicate an unexpected link between ALS and prion diseases, which both involve protein aggregation. The pathology associated with ALS is not believed to arise as a consequence of the inactivation of Sod1, but misfolding and aggregation of the Sod1 protein itself are thought to cause downstream neurotoxic events. Loss of Sod1 activity may not directly contribute to the pathology of ALS, but it may have additional pathological effects via increasing the frequency of the sporadic *de novo* formation of spongiform neuropathies.

## Experimental procedures

### Yeast strains and plasmids

The wild‐type strain 74D‐694 (*MATa ade1–14 ura3–52 leu2–3*, *112 trp1–289 his3–200*) and its isogenic derivatives deleted for *TSA1* (*tsa1*::*LEU2*) and *TSA2* (*tsa2*::*kanMX*) have been described previously (Sideri *et al*., 2010). Strains deleted for *SOD1* (*sod1*::*TRP1*), *SOD2* (*sod2*::*HIS*), *CTT1* (*ctt1*::*TRP1*) and *CTA1* (*cta1*::*HIS3*) were constructed in 74D‐694 using standard yeast methodology. Sup35 was tagged at its C‐terminus in with a tandem affinity purification (TAP) tag and has been described previously (Sideri *et al*., 2011). Overexpression of methionine sulphoxide reductase (*MSRA*) was achieved using a *GAL1*‐*MSRA*‐*GST* plasmid (pEGH) supplied by Open Biosystems. This plasmid expresses the *MXR1* gene encoding methionine S‐sulphoxide reductase.

### Growth and stress condition

Strains were grown at 30°C with shaking at 180 rpm in rich yeasts extract peptone dextrose (YEPD) medium (2% w/v glucose, 2% w/v bactopeptone, 1% w/v yeast extract) or minimal SD (0.67% w/v yeast nitrogen base without amino acids, 2% w/v glucose) supplemented with appropriate amino acids and bases. SGal media contained 2% w/v galactose and SRaf media contained 2% w/v raffinose in place of glucose. Media were solidified by the addition of 2% (w/v) agar. Strains were cured by three rounds of growth on YEPD agar plates containing 3 mM guanidine hydrochloride (GdnHCl).

### Determination of spontaneous [*PSI* ^+^] prion formation

A plasmid containing an engineered *ura3–14* allele, which contains the *ade1–14* nonsense mutation in the wild‐type *URA3* gene, was used to score the frequency of [*PSI* ^+^] prion formation (Manogaran *et al*., 2006). Briefly, freshly grown cells were inoculated into 25 ml of media selective for the plasmid (SD minus Leu) in 250 ml flasks and incubated with vigorous aeration for 16 h. Cells were washed and appropriate dilutions were plated on selective plates. A number of viable colonies were scored on plates selective for the plasmid (SD minus Leu) after 3 day incubation at 30°C. [*PSI* ^+^] prion formation was scored by growth on SD minus uracil following incubation at room temperature for 3 weeks (Manogaran *et al*., 2006). For anaerobic conditions, plates were incubated in an anaerobic jar (Oxoid) containing a gas generating kit (anaerobic system BR38; Oxoid). Each colony was spotted and scored as [*PSI*+] if it was able to grow on plates selective for [*PSI^+^*] but not in the presence of GdnHCl. The number of non‐prion derivatives was estimated by growing 200 of the resulting colonies on selective plates containing GdnHCl. The absence of growth indicates that it was caused by prion formation. Each experiment was performed at least in triplicate.

### Visualization of aggregation of prion proteins

*De novo* [*PSI* ^+^] prion formation was visualized as described previously using *CUP1‐SUP35NM‐GFP* (Sideri *et al*., 2011). Cells were grown for 16 h in SD minimal media, diluted to OD_600_ 0.1–0.2 and 50 μM copper sulphate added for induction of the *CUP1* promoter and visualized using an Olympus wide‐field microscope and MetaVue software (Bioimaging Facility, Faculty of Life Science, University of Manchester).

### Protein analysis

The analysis of Sup35 amyloid polymers by SDD–AGE was performed as described previously (Alberti *et al*., 2010). Sup35‐TAP was performed as described by Sideri *et al*. (2011). Methionine oxidation of Sup35 was detected using αMetO antibodies (Novus Biologicals).

### Electron microscopy

Samples for EM were prepared using the super quick freeze substitution method described previously (McDonald and Webb, 2011). High pressure freezing was performed using a Baltec HPM‐010 high‐pressure freezer. Samples were embedded in LR White resin, 70 nm sections cut on a Reichert Ultracut microtome and observed using an FEI Tecnai 12 Biotwin microscope at 100 kV acceleration voltage. Images were acquired using a GATAN Orius SC1000 CCD camera.
